# On the origin of orphan hybrids between *Aquilegia formosa* and *Aquilegia flavescens*

**DOI:** 10.1093/aobpla/ply071

**Published:** 2018-11-19

**Authors:** Jeffrey S Groh, Diana M Percy, Curtis R Björk, Quentin C B Cronk

**Affiliations:** Department of Botany, University of British Columbia, Vancouver, British Columbia, Canada

**Keywords:** *Aquilegia*, genetic swamping, herbarium, hybridization, introgression, range boundaries

## Abstract

We report the investigation of an *Aquilegia flavescens* × *A. formosa* population in British Columbia that is disjunct from its parents—the latter species is present locally but ecologically separated, while the former is entirely absent. To confirm hybridity, we used multivariate analysis of floral characters of field-sampled populations to ordinate phenotypes of putative hybrids in relation to those of the parental species. Microsatellite genotypes at 11 loci from 72 parental-type and putative hybrid individuals were analysed to assess evidence for admixture. Maternally inherited plastid sequences were analysed to infer the direction of hybridization and test hypotheses on the origin of the orphan hybrid population. Plants from the orphan hybrid population are on average intermediate between typical *A. formosa* and *A. flavescens* for most phenotypes examined and show evidence of genetic admixture. This population lies beyond the range of *A. flavescens*, but within the range of *A. formosa*. No pure *A. flavescens* individuals were observed in the vicinity, nor is this species known to occur within 200 km of the site. The hybrids share a plastid haplotype with local *A. formosa* populations. Alternative explanations for this pattern are evaluated. While we cannot rule out long-distance pollen dispersal followed by proliferation of hybrid genotypes, we consider the spread of an *A. formosa* plastid during genetic swamping of a historical *A. flavescens* population to be more parsimonious.

## Teaser

The outcomes of natural hybridization are incredibly varied, ranging from hybrid speciation to extinction of parental taxa. This research describes the investigation of an ‘orphan’ hybrid population between *Aquilegia formosa* and *Aquilegia flavescens*, two western North American taxa, in which the latter species is absent both locally and regionally. We present analyses of floral morphology, nuclear admixture and maternally inherited plastid sequence to confirm hybridity and discuss possible origin scenarios. We suggest that *A. flavescens* may have formerly occupied the region and became extirpated through genetic assimilation by its congener.

## Introduction

Orphan hybrid populations or lineages are those that occur in the absence of the parental taxa, presenting a challenge for understanding their origins. They may arise through dispersal beyond the range of the parental taxa. For example, the hybrid species *Senecio squalidus* occurs in the UK without either parent due to human-mediated dispersal of germplasm from a hybrid zone between two species in Sicily (Crisp 1972; James and Abbott 2005). On the other hand, hybrid populations may become orphaned by driving the disappearance of their progenitors from the landscape (Ellstrand and Elam 1993; Levin *et al.* 1996; Huxel 1999; Wolf *et al.* 2001; Todesco *et al.* 2016). This may occur by assimilation of parental genomes with continued crossing to fertile hybrid individuals (i.e. genetic swamping), which is expected when hybrid fertility is not reduced, and initial population sizes of parental taxa are small (reviewed in Todesco *et al.* 2016). Separately, hybrids could actively supplant parental populations through competitive exclusion, which may be facilitated by vegetative or parthenogenetic reproduction in hybrids. This phenomenon has been offered as an explanation for the orphan nature of disjunct hybrid populations of *Narcissus* × *perezlarae* (Marques *et al.* 2010) and the stick insect genus *Acanthoxyla* (Trewick *et al.* 2008). In addition, exclusion of progenitor lineages may be environmentally contingent, particularly in habitats that are marginal for either of the parents, or intermediate between their respective environmental optima (Anderson 1948).

In North American *Aquilegia* (commonly known as columbines), interspecific hybridization is a striking evolutionary phenomenon. Throughout their natural distribution, intergrading floral forms often occur in zones of species range overlap (Payson 1918; Munz 1946; Grant 1952; Whittemore 1997). Members are characteristically interfertile, and F_1_ hybrids often show high pollen fertility (Taylor 1967). Moreover, recent genomic study has implicated hybridization as a cause for extensive allele sharing in this group (Filiault *et al.* 2018). Previous workers have extensively studied hybrid populations of *Aquilegia formosa* × *A. pubescens* in the Sierra Nevada, CA, USA, demonstrating that divergent floral morphologies contribute to assortative mating through floral isolation (Grant 1952; Hodges and Arnold 1994; Fulton and Hodges 1999). Nonetheless, isolation is incomplete, contributing to a semipermeable species boundary between these species. While naturally occurring hybrids between other *Aquilegia* members are known, population-level studies of natural hybridization in the genus have thus far focused nearly exclusively on this species pair, with comparably little attention given to variable outcomes of hybridization among other species (but see Pelton 1957; Miller 1978).

**Figure 1.**
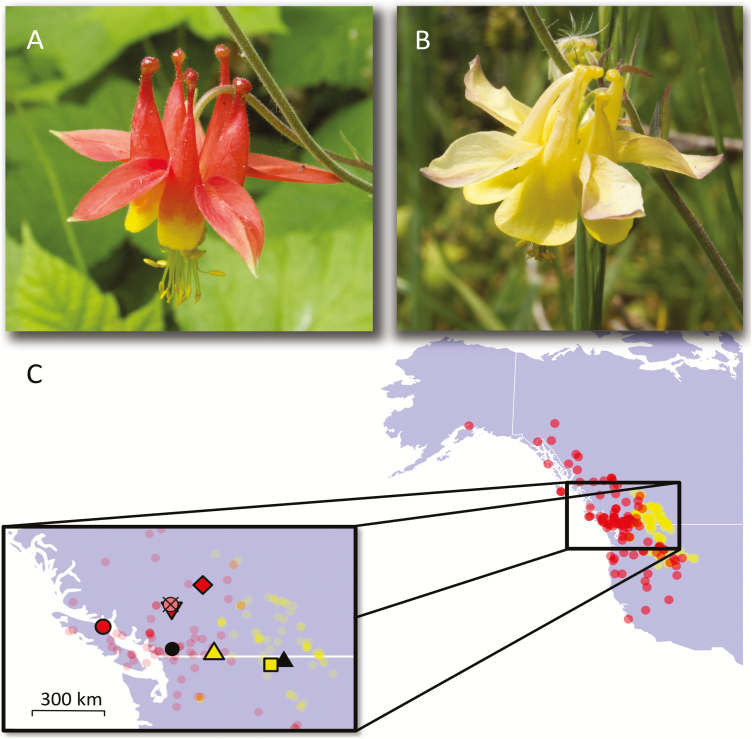
Flowers of *Aquilegia formosa* and *A. flavescens*, their natural ranges and field sampling sites. (A) Flower of *A. formosa* from near Clearwater, BC; (B) flower of *A. flavescens* from Mt. Kobau, BC; (C) range distributions of *A. formosa* (red) and *A. flavescens* (yellow) shown by geolocations of 191 herbarium specimens from University of British Columbia and University of Idaho. (Inset) Locations of field sites. Red circle, Robert’s Lake; red diamond, Clearwater; red upside-down triangle, Pavilion Clinton Highway; yellow triangle, Mt. Kobau; yellow square, Beehive Lakes; pink crossed circle, Marble Range; black circle and triangle, geographic range centroids calculated from herbarium specimen coordinates (see Materials and Methods) for *A. formosa* and *A. flavescens*, respectively. Herbarium specimen geolocations of *A. formosa* and *A. flavescens* are indicated in the background with transparent red and yellow circles, respectively.

The range of *A. formosa* (Fig. 1A) overlaps with that of its close relative, *A. flavescens* (Fig. 1B), (Payson 1918; Fior *et al.* 2013), in montane parts of western North America (Fig. 1C). The former species occurs commonly in lowlands along the Pacific slope, whereas the latter is most commonly restricted to high elevations of the Rocky Mountains. In mountainous regions where they occur in proximity, the species are generally separated altitudinally, with *A. flavescens* growing at high elevations and *A. formosa* growing along creek sides in valleys (Payson 1918, personal observation). Both species, diploids, are classified as hummingbird-pollinated (Grant 1994), and are also readily visited by bees and other insects which may act as pollinators. Differences in floral morphology and colour may potentially provide some floral isolation, but shared animal pollinators evidently effect cross-pollination, as hybrids form readily and persist in contact zones (Payson 1918; Grant 1952; Griffiths and Ganders 1983; Whittemore 1997). Intriguingly, botanists have noted a tendency for these hybrids to replace the typical parental forms. MacBride and Payson (1917) wrote: *‘One such state* [hybridity] *has been evolved in central Idaho and there in many localities entirely replaces the typical form of the species, so apparently it has acquired a certain degree of stability. This form is similar to A. flavescens except that the sepals are salmon-color or flushed with pink. This color modification is striking and extremely beautiful, well worth, it would seem, varietal recognition’*. Yet, confusion exists as to whether plants with intermediate floral colour (often labelled *A. flavescens* var*. miniana*) are hybrids, or rather pink-flowered morphs of otherwise typical *A. flavescens* (Whittemore 1997). Classification of such individuals should ideally integrate genetic, morphological and biogeographical information.

**Figure 2.**
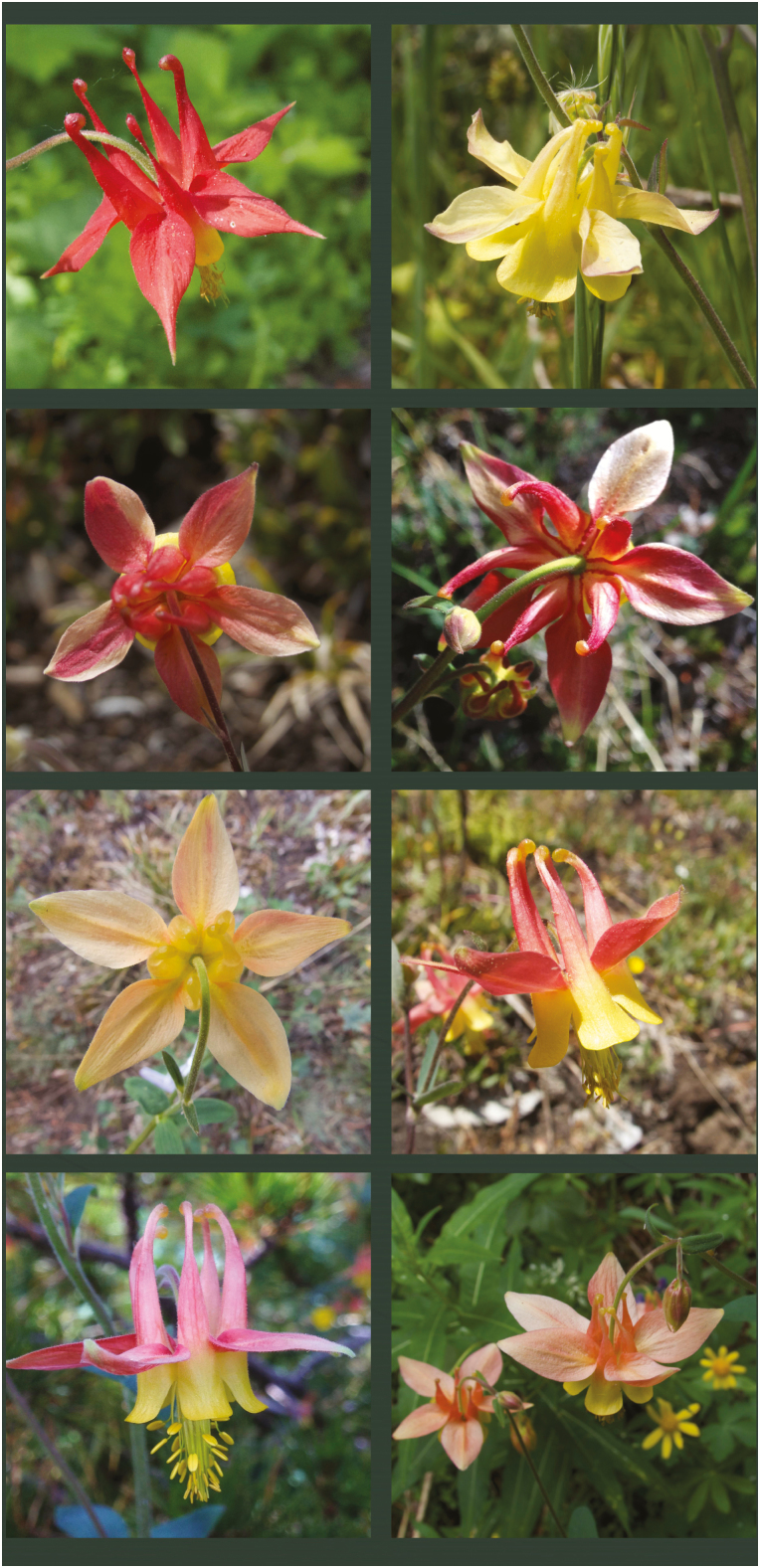
Flowers of *Aquilegia formosa*, *A. flavescens* and their hybrids. Top left: *A. formosa* flower from near Clearwater, BC; top right: *A. flavescens* flower from Mt. Kobau, BC; below: *A. flavescens* × *A. formosa* flowers from the Marble Range, BC.

A putative hybrid population of *A. flavescens* × *A. formosa* (Fig. 2) was originally identified by one of us (C.R.B.) on the upper slope of Porcupine Ridge in the Marble Range, British Columbia (BC) in the summer of 2016, and examined by the authors in detail the following summer. This site lies squarely within the typical range of *A. formosa*, but over 200 km to the west of the nearest reliable records of *A. flavescens*. Although hybrid populations appear to be common in south-eastern BC, this population is striking due to the absence of one of the parents. Two independent surveys of the surrounding area in different years failed to detect any pure *A. flavescens* individuals. We therefore sought to confirm the hybrid ancestry of this population, and to test hypotheses on the origin of the hybrid population. To confirm hybrid ancestry, we considered phenotypic and genetic (microsatellite) data of the putative hybrids in relation to allopatric parental populations. To assess the direction of hybridization and test hypotheses on the origin of the hybrid population, we compared maternally inherited plastid haplotypes of the hybrid individuals to those of allopatric parental populations as well as local *A. formosa* populations. As *Aquilegia* seeds do not disperse far, an *A. flavescens* maternal origin would support the extirpation of a pre-existing *A. flavescens* population, possibly through genetic swamping by local *A. formosa*. Alternatively, an *A. formosa* maternal origin could support spread of an *A. formosa* plastid lineage through a contact zone, or long-distance pollen dispersal from *A. flavescens*. Lastly, we used spatial analysis of herbarium specimen phenotypes to explore evidence for introgression throughout the distribution of these species.

## Materials and Methods

### Museum collections sampling

We measured floral phenotypes of 191 herbarium specimens from the University of British Columbia (UBC) and University of Idaho (ID) Stillinger herbaria. University of British Columbia specimens were measured directly from herbarium sheets; ID specimens were measured via size-scaled photographs accessed through the Consortium for Pacific Northwest Herbaria specimen database (http://www.pnwherbaria.org/data.php, 31 June 2017). Measurements focused on known defining species traits and other relevant traits revealed through preliminary inspection of specimens. For each pressed specimen, a single post-anthetic (open) flower was measured for seven continuous traits: corolla width, spur length, anther exsertion, petal lamina length, petal lamina width, sepal length and sepal width **[see****Supporting Information—Appendix S1****]**. Whether the petal laminae were cleft was recorded as a categorical trait. Herbarium specimens were geolocated according to information from herbarium sheet labels, the BC Geographical Names database (http://apps.gov.bc.ca/pub/bcgnws/, 31 June 2017) and Google Earth. To explore whether floral morphology corresponds to reported pollination mode, we measured the bill lengths of 10 rufous hummingbird (*Selasphorus rufus*) and 10 calliope hummingbird (*Selasphorus calliope*) specimens from the UBC Beaty Biodiversity Museum collection, which are the most common hummingbird species with breeding ranges overlapping the ranges of *A. formosa* and *A. flavescens*.

### Field sampling

#### Sites

Field sites included allopatric populations of *A. formosa* and *A. flavescens*, the putative hybrid population, and additional *A. formosa* sites in the vicinity of the hybrid population. We visited two allopatric populations of *A. formosa* at Robert’s Lake, Vancouver Island (50.22°N, 125.55°W), and near Clearwater, BC (51.83°N, 120.05°W), and two allopatric populations of *A. flavescens* on Mt. Kobau, BC (49.11°N, 119.67°W) and at Beehive Lakes, Idaho (48.66°N, 116.65°W) (Fig. 1C). The habitat of *A. formosa* sampling sites consisted of mesic roadsides and streambanks. *Aquilegia flavescens* sampling sites consisted of talus rockslide (Beehive Lakes) and montane sagebrush community (Mt. Kobau). The hybrid population was found between 1900 and 2100 m in elevation on an east-facing drainage beneath Porcupine Ridge in the Marble Range (51.11°N, 121.83°W). The *Aquilegia* at this site (see Fig. 2) occurred amongst patches of open dwarf tree-line forest interspersed with species-rich herbaceous vegetation, and were thus likely sheltered from the high winds experienced along the alpine ridge. We sampled tissue from two additional *A. formosa* sites in the vicinity of the hybrid population: one in an adjacent valley (51.08°N, 121.81°W), and one along the Pavilion-Clinton Highway, ~20 km from the hybrid population (50.97°N, 121.78°W). Populations were visited during peak bloom season in the summers of 2017 and 2018, with the exception of the population at Beehive Lakes, in which only several plants remained in bloom, precluding collection of floral phenotype data.

#### Sampling protocol

Plants with at least one post-anthetic flower were sampled in a haphazard fashion at least 1 m apart within the allopatric populations. In the hybrid population we sampled additional plants at the extremes of the present colour variation (ranging from nearly all yellow with pink tinge to completely red) to assess whether parental-type colouration correlated with parental-type morphology. For each plant, we photographed the outer face of the sepal whorl for a single flower using a Canon PowerShot sx50 HS digital camera (Canon Inc., Tokyo, Japan). A second photo was taken of the SpyderCheckr 24 colour card (Datacolor™, Lawrenceville, NJ) under the same light conditions for later standardization. We extracted nectar from one or multiple flowers from each plant with glass capillary tubes inserted into the back of the floral nectaries and measured nectar volume in the capillary tube before extruding it onto a percent sugar refractometer (Bellingham and Stanley Ltd, Tunbridge Wells, UK) for measurement of sugar concentration (weight % of sucrose equivalents in water). Samples of leaf tissue from each plant were collected in silica gel, and whole plants were then pressed in the field.

### Molecular methods

#### DNA extraction and amplification

We extracted DNA according to a 2 % CTAB extraction protocol (Doyle and Doyle 1987). Microsatellite loci for *Aquilegia* that are thought to evolve neutrally were chosen from the literature (Yang *et al.* 2005; Schlautman *et al.* 2014), and 11 were selected for genotyping a total of 72 individuals across five populations (see Table 1 for locus summaries and Table 2 for population summaries). We tested various non-coding plastid sequences for species-specific differences, and two loci were selected for further sequencing. The *trnT*-*trnL* region (GenBank: MK228981–MK228983) was amplified with primers **trnT**^**UGU**^**F** and **5′trnL**^**UAA**^**R** (Taberlet *et al.* 1991), and the *rps16* region (GenBank: MK228978–MK228980) was amplified using primers **rps16F** and **rps16R** as described in Shaw *et al.* (2005). We called microsatellite allele lengths using the software PeakScanner™ (Applied Biosystems™, Foster City, CA) and analysed the plastid sequence chromatograms using the software Sequencher™ (Gene Codes Corporation, Ann Arbor, MI).

**Table 1.** Summary statistics of microsatellite loci amplified from *Aquilegia formosa* and *A. flavescens.* Locus names refer to those given in Schlautman *et al.* (2014) with the exception of 11-20.1 which comes from Yang *et al.* (2005). *H*_o_, observed heterozygosity; *H*_e_, expected heterozygosity.

Locus	Motif	Allele length (bp)	No. of alleles	*H*_o_	*H*_e_
DR922072.1	GAA	200–227	10	0.78	0.8
DR945073.1	CCA	266–302	13	0.57	0.78
JZ009091.1	CAC	302–326	7	0.53	0.60
ER973157.2	GA	172–208	16	0.79	0.91
DR912270.1	AAG	206–233	10	0.70	0.81
ER969526.2	AT	302–364	24	0.41	0.92
DT741717.1	AAG	400–433	10	0.30	0.80
ER940655.2	TC	199–319	22	0.65	0.86
DR951797.1	GA	308–360	27	0.68	0.95
ER939871.2	AAG	137–179	13	0.97	0.81
11-20.1	CACAA	89–119	6	0.67	0.75
		Mean	**14.4**	**0.64**	**0.82**

**Table 2.** Population genetic summary statistics from 11 microsatellite loci amplified from *Aquilegia formosa* and *A. flavescens*. ^1^H is Shannon entropy, an information-based measure of allelic diversity in a population. *H*_o_, observed heterozygosity; *H*_s_, Nei’s gene diversity; *F*_IS_, inbreeding coefficient.

Species	Site	Sample size	^1^H	*H*_o_	*H*_s_	*F*_IS_
*A. flavescens*	Mt. Kobau	11	1.71	0.65	0.72	0.11
*A. flavescens*	Beehive Lakes	12	2.01	0.61	0.68	0.12
*A. formosa*	Robert’s Lake	6	1.70	0.53	0.59	0.17
*A. formosa*	Clearwater	14	1.94	0.64	0.72	0.12
Hybrid	Marble Range	29	1.90	0.59	0.72	0.16

### Analytical methods

#### Biometric analysis

Measurements of floral characters of herbarium specimens were analysed with linear discriminant analysis (LDA) implemented in the R package *MASS* (Venables and Ripley 2002). We used 5-fold cross-validation as a way of assessing the predictive accuracy of the discriminant function. In each iteration of this procedure, the data were first randomly subset into five groups, and the LDA model was fit on each of four training subsets, each time using the omitted portion as a prediction data set and averaging the misclassification rate over all five models. This procedure was repeated 1000 times, and the overall mean error rate was recorded. The same analysis and cross-validation procedures were repeated for field-collected specimens. In the latter analysis, the discriminant function was fitted and cross-validated using a data set containing only parental-type specimens. This model was subsequently used to classify the phenotypes of putative hybrids with equal prior probabilities of group membership.

#### Colour analysis

Images were shot in RAW format and imported into Lightroom™ (Adobe Systems Inc., San Jose, CA). Using SpyderCheckr colour correction software (Datacolor™, Lawrenceville, NJ), images of the colour card were used to create calibrations that were then applied to corresponding images of sepals in Lightroom. These calibrated images were then imported into Photoshop™ (Adobe Systems Inc., San Jose, CA) in the Adobe RGB colour space. The magnetic lasso tool was used to select a randomly chosen sepal or all sepals from each flower. We recorded the mean pixel value for each channel (red, green, blue) in RGB colour space from this selection, and used the ratio of the mean pixel values of the red and green channels as a measure of the sepal colour variation as perceived by humans (Bergman and Beehner 2008). The RGB model is an additive colour space in which the combination of red and green specifies yellow. Thus, the relative values for red and green channels capture the human-visible variation between red and yellow. The ratio was centred around zero by log-transformation (log R/G). For a simple validation of this method, we assigned images of hybrid specimens, which were highly variable in colour, an integer colour value from one to five to represent floral colour along the yellow-to-red axis. We then calculated the Pearson correlation coefficient between the log R/G scores and the visually assigned integer colour scores.

#### Genetic analysis

For each microsatellite locus, we recorded the overall allele size range (bp), number of alleles, and observed and expected heterozygosity (*H*_o_ and *H*_e_). Within populations, we calculated the average *H*_o_, gene diversity (*H*_s_) and inbreeding coefficient (*F*_IS_) using the R package *hierfstat* (Goudet 2005), and Shannon entropy in the *poppr* package (Kamvar *et al.* 2014) (see Table 1). Bruvo’s genetic distance, which assumes a symmetrical geometric model of microsatellite mutation (Bruvo *et al.* 2004), was calculated between all genotypes in *poppr*. To ordinate genotypes, we implemented principal coordinate analysis with a correction for negative eigenvalues on the Bruvo’s distance matrix using the R package *ape* (Paradis *et al.* 2004). We constructed distance-based trees in the R packages *ape* and *phangorn* (Schliep 2011) using neighbour joining (NJ) (Saitou and Nei 1987) and unweighted paired group method with arithmetic mean (UPGMA) (Sokal and Michener 1958) algorithms.

To directly test for admixture, we applied a distance-based multivariate procedure, discriminant analysis of principal components (DAPC), which is implemented in the R package *adegenet* (Jombart 2008; Jombart *et al.* 2010). In this procedure, scaled microsatellite frequencies are first projected onto orthogonal principal component axes, and a discriminant axis is constructed as a linear combination of principal component axes, which maximizes between-group variance while minimizing within-group variance. As in the morphometric analysis, we first constructed the model using only pure parental types. After optimizing the number of principal components to be retained in the analysis using the *a*-score (Jombart *et al.* 2010), we empirically determined the misclassification rate over 1000 iterations of the cross-validation procedure described above. Discriminant axis scores of the putative hybrids were then predicted according to the calculated model with equal prior probabilities of group membership.

As a parallel approach, we applied a Bayesian clustering model to the microsatellite data using the software STRUCTURE version 2.3.4 (Pritchard *et al.* 2000). As our aim was to test whether individuals from the Marble Range had hybrid ancestry, we implemented the analysis by setting genotypes of individuals from reference populations as predefined clusters and estimating the ancestry of the putative hybrids. This was done by setting ‘POPFLAG = 1’ for parental specimens, which were assigned to two different clusters, and ‘POPFLAG = 0’ for putative hybrids, and specifying ‘update allele frequencies using only individuals with POPFLAG=1’ in the program’s front end. We first ran a standard analysis using only a data set consisting of parental specimen genotypes without prior information to verify correct species separation with *K* (the number of genetic clusters) set equal to two. All STRUCTURE runs used the admixture model allowing for correlated allele frequencies, with 100 000 burn-in and Markov chain Monte Carlo repetitions. Results were checked for consistency across 20 runs and final ancestry coefficients of individuals were averaged using the program CLUMPP (Jakobsonn and Rosenberg 2007).

Plastid sequences were concatenated after alignment with MUSCLE (Edgar 2004) and used to construct a statistical parsimony network using the R package *haplotypes* (Aktas 2015), scoring indels according to the simple indel coding method (Simmons and Ochoterena 2000).

#### Geographic analysis of phenotypic variation

To investigate evidence for morphological introgression across the distribution of *A. formosa* and *A. flavescens*, we assessed whether the extent of phenotypic discrimination of herbarium specimen phenotypes was negatively associated with geospatial proximity to the range centroid of the alternative species, relative to proximity to the centroid of the same species. To calculate range centroids, we first converted latitude, longitude coordinates into radians (ϕ,θ) by multiplying by $\frac{\pi }{180}$. Next, assuming a spherical Earth, coordinates were converted to a Cartesian basis according to the transformation:

x=cosθcosϕ ​

y=cosϕsinθ

z=sinϕ

Cartesian position vectors were averaged across specimens for each species and converted back to latitude and longitude coordinates according to the transformation:

$$latitude=\;\frac{180}{\pi }arcsin\frac{z}{\sqrt{x^{2}+y^{2}+z^{2}}}$$

$$longitude=\;\frac{180}{\pi }arctan\frac{y}{x}.\text{​​}$$

For each geolocated herbarium specimen, distances from species centroids were calculated using the *distHaversine* function in the R package *geosphere*, which also assumes a spherical Earth (Hijmans 2016). We used the natural logarithm of the ratio of distance from conspecific and heterospecific centroids to reflect spatial proximity to the range of the alternative species relative to the range of the same species.

## Results

### Interspecific and hybrid variation

#### Distribution and flowering time

Geolocations of herbarium specimens confirmed that the ranges of the parental species overlap in montane regions west of the Rocky Mountains Fig. 1C), and that they tend to occur at different altitudes **[see****Supporting Information—Appendix S2****]**. However, altitudinal separation is not strict; while *A. flavescens* mainly occurs at elevations >1000 m, *A. formosa* has a wider altitudinal range, and is common at—but not restricted to—lower elevations. Note that while these data reflect the known biology and range information of the species, geolocation uncertainty may contribute to substantial altitude measurement error for data points in mountainous regions. Analysis of collection dates, a proxy for flowering time, revealed broad overlap in flowering time between species **[see****Supporting Information—Appendix S2****]**. After correcting for covariance with altitude, the intercept difference was not significantly different from zero (*F*_1, 169_ = 0.31, *P* = 0.58).

#### Sepal colour

**Figure 3.**
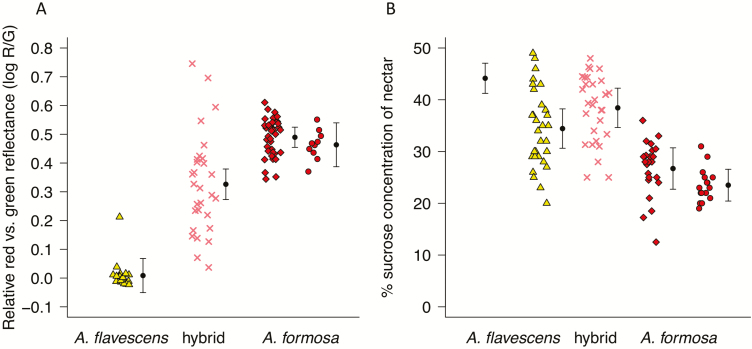
Comparison of floral phenotypes of plants from the Marble Range, BC, with allopatric *Aquilegia formosa* and *A. flavescens*. (A) Colour reflectance of outer sepal whorl. Yellow triangles, *A. flavescens* from Mt. Kobau, BC; pink crosses, *A. flavescens* × *A. formosa* hybrids from the Marble Range; red diamonds, *A. formosa* from near Clearwater; red circles, *A. formosa* from Robert’s Lake, BC. (B) Nectar concentration. Far left: data for *A. flavescens* from Bacon (2010); otherwise, symbols are the same as in (A). Error bars represent 95 % CIs.

Colour scores (log R/G) were highly correlated with visually assigned integer scores within the colour-variable hybrids (*r* = 0.85), validating the use of the log R/G metric. Field-collected plants from parental populations exhibited non-overlapping variation in the relative amount of red vs. green reflectance in the sepals (Fig. 3A). However, a single pink-flowered individual was found in the population of *A. flavescens* on Mt. Kobau; the rest of the individuals in the population were uniformly yellow-flowered. While this individual had a log R/G score that overlapped with those of the hybrids, it clustered with *A. flavescens* in both morphometric and genetic analyses. With respect to the parental populations, colour variation was on average intermediate but highly variable in the hybrid population. The range of colour variation in the hybrids exceeded the extent of red reflectance seen in our samples of pure *A. formosa*, although no flowers were as yellow (green-reflectant) as those of pure *A. flavescens* (Fig. 3A).

#### Floral form

The floral morphology discriminant axis scores of herbarium specimens showed distinct yet overlapping distributions. The mean classification error rate over 1000 iterations of 5-fold cross-validation was 0.08, in accordance with some degree of visual overlap of the distributions for each species along the discriminant axis. The loadings of each character onto the discriminant axis (see Table 3) indicate their relative contribution to the discrimination of species. The dimensions of the laminae showed the highest loadings onto the discriminant axis. The functional significance of this trait may relate to exclusion of insect pollinators. Recurved or short petals preclude flying insects from landing in several bird-pollinated species (Cronk and Ojeda 2008). Furthermore, whether the petal laminae were cleft proved to be a useful discriminator between *A. formosa* and *A. flavescens* flowers in practice. The proportion of individuals with cleft laminae differed notably between species (*A. formosa*: proportion (*p*) = 0.37, 95 % confidence interval (CI) = 0.28, 0.47; *A. flavescens*: *p* = 0), such that the presence of cleft petal laminae is diagnostic of *A. formosa.*

**Table 3.** Floral trait summary statistics for *Aquilegia* specimens from the University of British Columbia (UBC) and University of Idaho (ID) Stillinger herbaria. SE, standard error; LDA loading, weighting of trait in the linear discriminant function which distinguishes between *A. formosa* and *A. flavescens* floral phenotypes.

Floral trait	Species	Mean (cm)	Sample size	SE	95 % CI		LDA loading
					Lower	Upper	
Lamina length	*A. formosa*	0.31	95	0.01	0.29	0.33	−2.73
	*A. flavescens*	0.58	81	0.02	0.55	0.61	
Lamina width	*A. formosa*	0.39	92	0.01	0.37	0.41	−2.27
	*A. flavescens*	0.65	80	0.02	0.62	0.68	
Corolla width	*A. formosa*	1.45	90	0.03	1.40	1.51	−2.02
	*A. flavescens*	1.88	80	0.04	1.80	1.95	
Spur length	*A. formosa*	1.74	93	0.03	1.68	1.81	1.77
	*A. flavescens*	1.48	78	0.03	1.42	1.54	
Sepal width	*A. formosa*	0.74	98	0.02	0.71	0.78	1.36
	*A. flavescens*	0.72	75	0.02	0.67	0.77	
Anther exsertion	*A. formosa*	1.11	96	0.03	1.06	1.16	1.19
	*A. flavescens*	0.68	79	0.02	0.64	0.72	
Sepal length	*A. formosa*	2.11	97	0.04	2.03	2.19	0.48
	*A. flavescens*	1.96	80	0.04	1.88	2.05	
Cleft laminae *(proportion)	*A. formosa*	0.37*	97	0.05*	0.28*	0.47*	−0.29
	*A. flavescens*	0*	83	0*			

**Figure 4.**
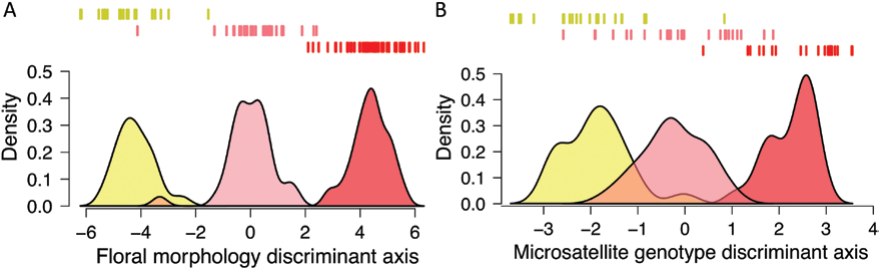
(A) Ordination of floral phenotypes of plants from the Marble Range, BC, along the discriminant axis that separates phenotypes of allopatric *Aquilegia formosa* and *A. flavescens*. Data are shown above y-axis. Yellow, *A. flavescens*; pink, *A. flavescens* × *A. formosa* hybrids; red, *A. formosa*. (B) Ordination of microsatellite genotype scores along the discriminant axis (from DAPC, see Materials and Methods) that best separates parental species genotypes. Colours are the same as in (A).

In contrast to the analysis of herbarium specimens, the LDA of field-collected allopatric specimens achieved clean interspecific separation of floral phenotypes (Fig. 4A). Furthermore, the misclassification rate was 3 × 10^−4^, considerably lower than that of the LDA of herbarium specimen phenotypes. The loadings of traits onto the discriminant axis were similar to those in the herbarium specimen analysis, with the dimensions of the laminae again having the largest loadings. The proportions of field-collected individuals with cleft petal laminae also closely matched those estimated from herbarium specimens (*A. formosa*: *p* = 0.38, 95 % CI = 0.27, 0.51; *A. flavescens*: *p* = 0). The field-collected parental specimens were thus taken to be representative of the ‘pure’ species floral phenotypes and were used thereafter for direct comparison with the putative hybrid phenotypes.The floral morphology discriminant axis scores of the Marble Range flowers fell largely in between the clusters of the representative parental types (Fig. 4A). The proportion of Marble Range individuals with cleft petal laminae was also intermediate between those of the parental species (*p* = 0.22, 95 % CI = 0.11, 0.39), although the 95 % CI for the difference in proportions between the Marble Range population and all *A. formosa* specimens marginally overlapped zero (95 % CI = −0.05, 0.37). Depending on the genetic basis of interspecific differences, the overall intermediacy of hybrid phenotypes could result from intermediacy in additive traits, or from mismatched combinations of non-additive traits from either parental species. We therefore investigated the distribution of each floral character independently in the Marble Range plants in relation to the parental trait distributions. This revealed that the Marble Range floral characters are on average intermediate for all continuous traits, with the exception of sepal length **[see****Supporting Information—Appendix S3****]**. These results imply an additive genetic basis in the traits governing interspecific differences between the parental species. As parental-type traits should segregate in recombinant hybrids, we also calculated pairwise trait correlations in the hybrids to investigate possible co-segregation of traits [**see Supporting Information—Appendix S4]**.

We found that the sepal colour of the Marble Range flowers was not predictive of morphological affinity to either parental type. On the contrary, the log R/G scores showed a weak negative correlation with the discriminant axis scores of the Marble Range flowers (*r* = −0.30, *P* = 0.11), although this result was insignificant.

#### Nectar

Mean nectar sugar concentration for *A. formosa* populations were 26.7 % (95 % CI = 22.7, 30.7) at Clearwater and 23.5 % (95 % CI = 20.4, 26.6) at Robert’s Lake, in agreement with a measure previously reported by Gut *et al.* (1977) of 25 % for this species (*N* = 94). For *A. flavescens* at Mt. Kobau, mean nectar sugar concentration was 34.4 % (95 % CI = 30.6, 38.2). For comparison, Bacon (2010) reported a nectar concentration of 44.15 % (95 % CI = 41.24, 47.06) for *A. flavescens.* In the hybrid population, mean nectar sugar concentration was 38.4 % (95 % CI = 34.6, 42.2), notably higher compared to the means of *A. formosa* populations, and similar to the estimates for *A. flavescens*. Based on available data (Fig. 3B), *A. formosa* seems to have consistently lower nectar concentration than *A. flavescens*, and the hybrids are more similar to the latter species for this trait.

Although we found up to 8 µL of nectar within a single *A. formosa* nectar spur, a large portion of flowers contained insufficient volumes to collect (<1 µL). Moreover, we observed indications of a high incidence of nectar robbing. At one locality, a visual survey of all 40 *A. formosa* flowers along a 50 m transect adjacent to a creek revealed that 35 % showed signs of lacerations to the nectaries, and several insect taxa were observed nectar robbing during the course of the study **[see****Supporting Information—Appendix S5****]**. Bacon (2010) reported a mean volume of 4.46 µL (95 % CI = 3.50, 5.42) for *A. flavescens* using the methods used here. All flowers sampled in the Marble Range population contained measurable quantities of nectar, with a mean of 3.48 µL (95 % CI = 2.67, 4.29). While these values give a general sense of nectar volumes in these populations, meaningful comparison of the hybrid and parental types for this phenotype remains inconclusive, due to the complex dependencies of nectar volume on developmental stage, environmental conditions and nectar removal by visitors.

### Interspecific genetic differentiation and the genetic signal of hybridity

#### Nuclear genotype

Analysis of microsatellite data revealed genetic differentiation between *A. formosa* and *A. flavescens*. In the absence of prior information, the STRUCTURE analysis of allopatric specimen genotypes was able to perfectly distinguish species when hybrid genotypes were excluded (not shown) and *K* was set equal to two. Unweighted paired group method with arithmetic mean and NJ trees also showed strong species clustering when hybrids were excluded (not shown). The genetic data performed more poorly at species discrimination as compared to floral morphology data; cross-validation showed that the DAPC model for allopatric parental specimens had a misclassification rate of 0.04 (vs. 3 × 10^−4^ for the morphology LDA). To see if this discrepancy could be a result of differences in sample size, we randomly subset the floral phenotype data set to contain the same sample sizes as in the genetic data set and repeated the cross-validation procedure. The classification error rate increased to 8 × 10^−4^, a value still considerably lower than the classification error rate based on genotype. This observation suggests that the species are more divergent in morphology than in genotype.

**Figure 5.**
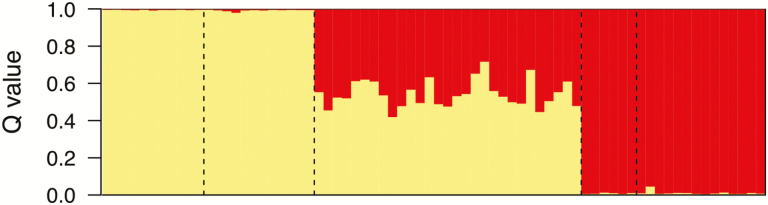
Ancestry coefficients (from STRUCTURE analysis of 11 microsatellite loci) of plants from the Marble Range, BC, show evidence for dual ancestry from *Aquilegia formosa* and *A. flavescens.* Yellow, *A. flavescens* genetic cluster; red, *A. formosa* genetic cluster. Populations are delimited by dotted lines. From left to right: Beehive Lakes, Mt. Kobau, Marble Range, Robert’s Lake, Clearwater. *Q-*values of parental specimens represent posterior probability of correct assignment while *Q-*values of hybrids are ancestry estimates.

All analyses performed suggested that the individuals from the Marble Range population have genetic ancestry from both *A. flavescens* and *A. formosa* (Figs 4B and 5). In the distance-based analysis, the scores of the hybrids tend to be intermediate between the expected values of each species (Fig. 4B). Replicate runs of STRUCTURE were highly consistent and showed evidence of admixture in the hybrids, with slightly greater estimated ancestry from *A. flavescens* than from *A. formosa* (mean 54 and 46 %, respectively). Hybrids also showed intermediate placement between parental clusters in the principal coordinate analysis based on Bruvo’s distance **[see****Supporting Information—Appendix S6****]** and showed variable placement in the NJ and UPGMA trees **[see****Supporting Information—Appendix S7****]**.

#### Plastid genotype

**Figure 6.**
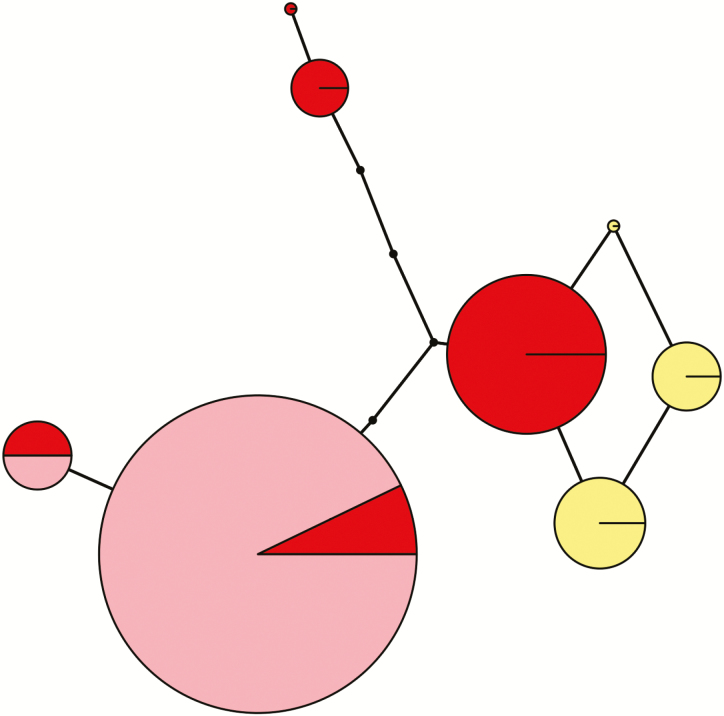
Statistical parsimony haplotype network constructed from concatenated plastid regions *rps16* and *trnT-trnL* indicates that *Aquilegia* hybrids from the Marble Range, BC, are descended from an *A. formosa* maternal lineage. Each coloured node represents a unique haplotype, with the area of the node proportional to the number of individuals possessing that haplotype. Yellow, *A. flavescens*, sample size = 15; red, *A. formosa*, sample size = 25; pink, *A. flavescens* × *A. formosa* hybrids from the Marble Range, BC, sample size = 29. Internodes represent the number of sequence differences between haplotypes. Haplotypes found in the hybrids are shared by local *A. formosa* populations.

No haplotypes constructed from plastid sequences were represented in members of both species (Fig. 6). A single 5-bp indel within *trnT*-*trnL*, which is treated as a single mutation in the haplotype network, was predominantly species-specific and contributed to species separation of haplotypes; however, a single yellow-flowered individual at Mt. Kobau notably possessed the *A. formosa* allele at this site. We searched GenBank records (Fior *et al.* 2013) and determined that the indel sequence is a 5-bp repeat with zero to three repeat units present in different members of the genus. *Semiaquilegia adoxoides*, sister to *Aquilegia* (Fior *et al.* 2013), possesses one unit, *A. jonesii* possesses zero, *A. formosa* and several congeners possess two, and *A. flavescens* alone possesses three out of the taxa represented, suggestive of a history of insertion and reversal at this locus.

We found that the hybrids possessed an *A. formosa*-type plastid haplotype (Fig. 6), which was shared only by nearby populations of *A. formosa* in the adjacent valley and 20 km away from the hybrid population along the Pavilion-Clinton Highway.

### Clinal variation in floral morphology of herbarium specimens

**Figure 7.**
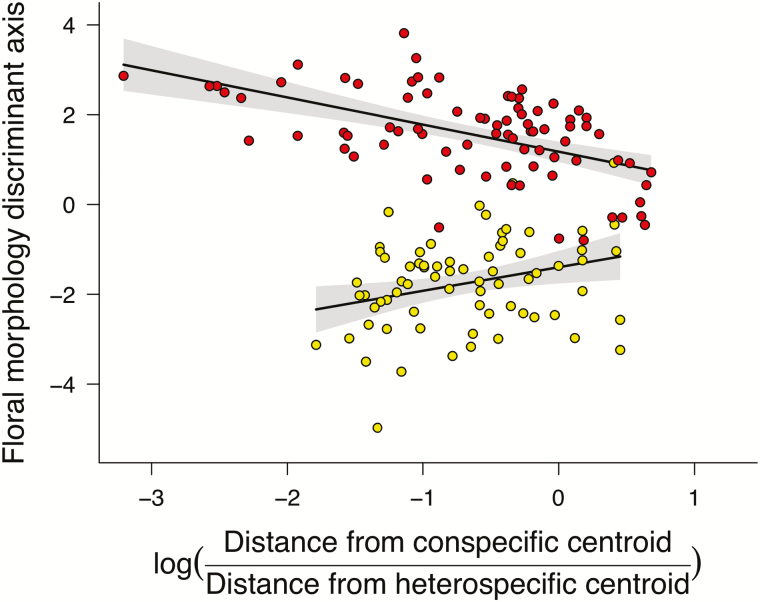
Clinal variation in floral phenotypes of herbarium specimens across the distribution of *Aquilegia formosa* and *A. flavescens* suggests morphological introgression on a broad geographic scale. Specimens located close to zero on the y-axis are less well phenotypically discriminated from the alternative species than those with higher absolute value. Positive values on the x-axis indicate closer proximity to the centroid of the alternative species, whereas negative values indicate closer proximity to the centroid of the same species. Red, *A. formosa*, sample size = 80; yellow, *A. flavescens*, sample size = 71.

We found a negative association between the extremes of floral morphology (higher absolute values along the floral morphology discriminant axis) and spatial proximity to the geographical range centroid of the alternate species relative to that of the same species (Fig. 7). Put another way, specimens which originate close to the range centre of the alternative species, and at the periphery of their own species’ range, on average more closely resemble the alternative species.

## Discussion

### Direction of the hybridization event producing the hybrid population

Given that the haplotype of the Marble Range hybrid plants was shared by *A. formosa* both in the adjacent valley and 20 km away along the Pavilion-Clinton Highway, the evidence supports the idea that the hybrids descended from a local *A. formosa* maternal lineage. Interestingly, an alternative *A. formosa* haplotype is shared by individuals in both Robert’s Lake and Clearwater, which occur on either side of the Marble Range, over 400 km apart. This does not support an isolation-by-distance pattern and likely reflects a complex phytogeographic history of this species in the mountainous and highly dissected landscape of BC.

As plastid inheritance appears to be maternal in several *Aquilegia* and in other members of Ranunculaceae (Corriveau and Coleman 1988), the haplotype analysis indicates that *A. flavescens* alleles were necessarily transmitted paternally during the formation of the hybrid population. While it is conceivable that there exist source populations of *A. flavescens* elsewhere in the Marble Range, there are no confirmed records of this species from this region of the province, and the nearest known populations are over 200 km to the east, suggesting that either (i) historical *A. flavescens* populations occurred in this region, or (ii) long-distance pollen dispersal occurred. Under the first scenario, the hybrids would represent ‘ghosts’ of one or more extirpated *A. flavescens* populations. Alternatively, under the second scenario, long-distance pollen dispersal could have resulted from hummingbird migrational movements. The ranges of both the rufous hummingbird and the calliope hummingbird overlap with the distributions of *A. formosa* and *A. flavescens*. Both species have been directly observed to visit *A. formosa* (Grant 1952; Chase and Raven 1975; Fulton and Hodges 1999; **see****Supporting Information—Appendices S5** and **S8**), and it is likely that hummingbirds visit *A. flavescens* as well (Grant 1994; Whittall and Hodges 2007; Bacon 2010). We directly observed rufous hummingbirds in the Marble Range and captured video footage of a visit to *Aquilegia* at one locality **[see****Supporting Information—Appendix S8****]**. The correspondence between the bill lengths of these hummingbirds and the spur lengths of the *Aquilegia* species **[see****Supporting Information—Appendix S9****]** further suggests they may cross-pollinate the latter, and they are well known to travel large distances during migration.

### Establishment of the hybrid population in the Marble Range

Either scenario, whether the extirpation of *A. flavescens* or whether *A. flavescens* sired this population from afar, raises questions. How did the hybrids become so numerous? And how was a high proportion of neutral *A. flavescens* ancestry maintained? Animal-mediated pollen dispersal over long distances is likely to be rare, and as such, gametophyte influx alone seems unlikely to have yielded such a large population. Furthermore, the STRUCTURE analysis did not indicate the presence of pure or backcrossed *A. formosa* individuals (Fig. 5), implying hybrids proliferated autonomously to some extent after the initial hybridization event. There are several explanations for how this might have occurred.

First, the habitat may have been previously unoccupied by either parental species, allowing for hybrids to colonize a vacant spatial niche at a higher elevation than a maternal *A. formosa* population. However, during the ascent to the ridge from the adjacent valley, we observed *A. formosa* up to elevations of 2100 m, and herbarium records demonstrate that this species inhabits high elevations with some frequency **[see****Supporting Information—Appendix S2****]**. Moreover, it is unlikely for the ancestral hybrid seeds to have landed far from maternal plants, as *Aquilegia* seeds possess no mechanism for long-distance dispersal, but are rather passively wind-dispersed from dehiscent follicles.

A second explanation, not mutually exclusive with the previous, is that hybrids competitively excluded a maternal population of *A. formosa* on the ridge. A complement of alleles from *A. flavescens*, a highland specialist, could have conferred a competitive advantage to hybrids. Indeed, the dry alpine habitat of the ridge appears more suitable for pure *A. flavescens* than for pure *A. formosa*, so it seems likely that *A. flavescens* alleles would confer a selective advantage in this habitat. Furthermore, while *A. formosa* was observed at comparable elevations on the other side of the ridge, the density of these individuals had notably decreased by this point, as had their stature, indicating that the habitat at this elevation was marginal for that species. However, as *A. flavescens* is not known from this region, the idea that an *A. formosa* population occupied the highland habitat prior to the establishment of the hybrid population invokes a long-distance pollen dispersal event from *A. flavescens* that is likely to be rare. Furthermore, under the long-distance dispersal hypothesis, the question of how a high proportion of neutral *A. flavescens* ancestry could have spread through the population is challenging to explain.

Alternatively, hybrids may have proliferated through genetic swamping of a pre-existing population of *A. flavescens* which inhabited the ridge upon secondary contact. Under this scenario, a larger *A. formosa* population size could have increased the likelihood of early-generation hybrids backcrossing to *A. formosa*, resulting in the probable spread of the *A. formosa* haplotype and the loss of an *A. flavescens* plastid lineage. Notably, an *A. formosa*-type plastic haplotype has been found in an introgressed population of *A. flavescens* in the Wenatchee Mountains, Washington State, USA (J. S. Groh, unpubl. data), supporting the plausibility of this hypothesis. Under the genetic swamping hypothesis, the high proportion of *A. flavescens* ancestry can be more easily reconciled, as genetic swamping does not lead to a loss of parental alleles, but only of intact parental genomes (Todesco *et al.* 2016).

### Classification practice obscures introgression on a broad geographic scale

How can we explain the discrepancy in species discrimination power between the analysis of herbarium specimens and that of collected allopatric populations? Quantitative analyses of floral morphology agree with visually discernable differences, confirming that true interspecific differences in floral morphology do exist. However, phenotypes of the ‘pure’ species are evidently two extremes of a continuum that arises from hybridization in contact zones throughout the distribution of these species. The dichotomous labelling of questionable specimens by taxonomists as one or the other species conceals the apparent reality that hybridization between *A. formosa* and *A. flavescens* occurs commonly where they come into contact, resulting in overlapping phenotype distributions. This showcases a conflict between the discreteness of taxonomy and the continuity of phenotypic variation. In his monograph of North American *Aquilegia*, Payson (1918) chose to retain the two forms as separate species, *‘since in the centers of their ranges [A.] formosa and [A.] flavescens are amply distinct’.* Yet, Payson encouraged us not to forget ‘*that in certain regions the two actually merge’.* Indeed, we uncovered a signal of clinal variation in floral morphology across the distribution of *A. formosa* and *A. flavescens* (Fig. 7). Such a cline likely reflects the effects of introgressive hybridization over a broad geographic scale.

### Conservation implications and alternative views on the dissolution of species boundaries

Where *A. formosa* and *A. flavescens* occur in regions of sympatry, the most significant barrier to gene flow is likely to be their altitudinal separation. Even so, the former has been shown here to occur with reasonable frequency at similar elevations to the latter. This barrier may be further attenuated under climate change if *A. formosa* undergoes an upward altitudinal range shift, as is predicted to occur in many plant species (Lenoir *et al.* 2008; Gómez *et al.* 2015). Under this scenario, locally isolated highland populations of *A. flavescens* could become threatened by genetic swamping through hybridization with their lowland congener.

An alternative outlook stresses the evolutionary potential afforded by hybridization. Biologists have increasingly come to recognize the enormous adaptive potential resulting from hybridization (e.g. Anderson 1949; Rieseberg and Wendel 1993; Arnold 2006; Arnold and Kunte 2017; Suarez-Gonzalez *et al.* 2018). Moreover, the role of hybridization as a source of evolutionary novelty has recently received enhanced attention in light of climate change; genetic introgression through interspecific hybridization may provide populations at risk with access to novel genetic variation sufficient for adaptation to changing climate conditions (Hamilton and Miller 2016). The extensive allele sharing through hybridization seen in North American *Aquilegia* (Filiault *et al.* 2018) may indeed be a crucial source of variation for natural selection to act upon in changing environments, especially considering the diversity of abiotic environments inhabited by members of the genus.

The dissolution of species boundaries in the Marble Range should not be regarded as a loss of diversity, but as an evolutionary resource. Pockets of hybridization between *A. formosa* and *A. flavescens* throughout their distribution likely act as harbours for adaptive genetic exchange among populations occurring in topographically diverse environments. Moreover, given the suggestive evidence for geographically widespread phenotypic introgression (Fig. 7), it seems likely that adaptive introgression between these two species has the potential to occur throughout their distribution.

In order to reconcile the traditional taxonomic treatment of these forms as distinct species with the concept of an expanded allele pool through hybridization, a more inclusive evolutionary unit than the biological species becomes useful. These members of *Aquilegia* belong to a syngameon, defined as a group of species interconnected by periodic or frequent genetic exchange (Grant 1981; Suarez-Gonzalez *et al.* 2018). They are further linked through hybridization to other western North American members of the genus. *Aquilegia formosa* hybridizes with *A. pubescens*, both *A. formosa* and *A. flavescens* hybridize with *A. caerulea*, *A. flavescens* hybridizes with *A. brevistyla*, and these congeners in turn participate in other hybridization events (Munz 1946; Grant 1952; Pelton 1957). The ramifications of this web of hybridization are only beginning to be explored using genomics (Filiault *et al.* 2018), and continued investigation in this group will greatly enhance our understanding of the intricate and varied evolutionary outcomes of interspecific hybridization. Our observations of hybridity in the Marble Range not only further elucidate the situation in *Aquilegia* but also point to a promising new system in which to explore hybridization between recently diverged species at the population level.

## Data

Data and original R script files for analysis are hosted at https://github.com/jgroh/aquilegia-hybrids.

## Sources of Funding

We are grateful for funding from a UBC Botany Work Learn International Undergraduate Research Award, a UBC Botany and Zoology Student Research Award and a Walter H. Lewis Award in Plant Diversity (all to J.S.G.), and from the Natural Sciences and Engineering Research Council of Canada (NSERC) Discovery Grants Program (grant no. RGPIN-2014-05820) to Q.C.B.C.

## Contributions by the Authors

J.S.G. and Q.C.B.C. planned the research. J.S.G. collected herbarium and field data, extracted DNA, performed analyses and wrote the manuscript. D.M.P. assisted with field work and generated molecular data. C.R.B. identified the population and carried out initial field surveys. Q.C.B.C. assisted with field work, suggested analyses, edited the manuscript and provided funding for the study. All authors contributed to and approved the final manuscript.

## Conflict of Interest

None declared.

## Supplementary Material

Supplementary Appendix

Supplementary Video

Supplementary Collections Blind

## Supporting Information

The following additional information is available in the online version of this article—

Appendix S1. Diagram of floral characters measured from pressed *Aquilegia* specimens.

Appendix S2. Linear regression of collection date (a proxy for flowering time) against altitude for herbarium specimens of *Aquilegia formosa* and *A. flavescens*.

Appendix S3. Distributions of seven continuous floral traits in allopatric *Aquilegia formosa* and *A. flavescens* and a hybrid population in the Mable Range, British Columbia (BC).

Appendix S4. Statistically significant pairwise floral trait correlations in *Aquilegia* hybrids.

Appendix S5. Photograph collage of visitors to flowers of *Aquilegia formosa* observed during the course of this study.

Appendix S6. Principal coordinate analysis implemented on a genetic distance matrix (Bruvo’s distance) of microsatellite genotypes of *Aquilegia formosa*, *A. flavescens* and hybrids from the Marble Range.

Appendix S7. Neighbour joining (NJ; left) and unweighted paired group method with arithmetic mean (UPGMA; right) trees constructed from a distance matrix (Bruvo’s distance) of microsatellite genotypes of *Aquilegia formosa*, *A. flavescens*, and hybrids from the Marble Range.

Appendix S8. Camera trap video of a female rufous hummingbird visiting flowers of *Aquilegia formosa.*

Appendix S9. Comparison of nectar spur lengths of *Aquilegia* with the bill lengths of two hummingbird species with overlapping ranges.
