# Supplementary material for: On the origin of orphan hybrids between Aquilegia formosa and Aquilegia flavescens
Source: AoB Plants. 2018 Nov 19;11(1):ply071. doi: 10.1093/aobpla/ply071 (PMC6341775; doi:10.1093/aobpla/ply071)
Supplement: Supplementary Appendix [file ply071_suppl_supplementary_appendix.docx]

**SUPPLEMENTARY MATERIALS**


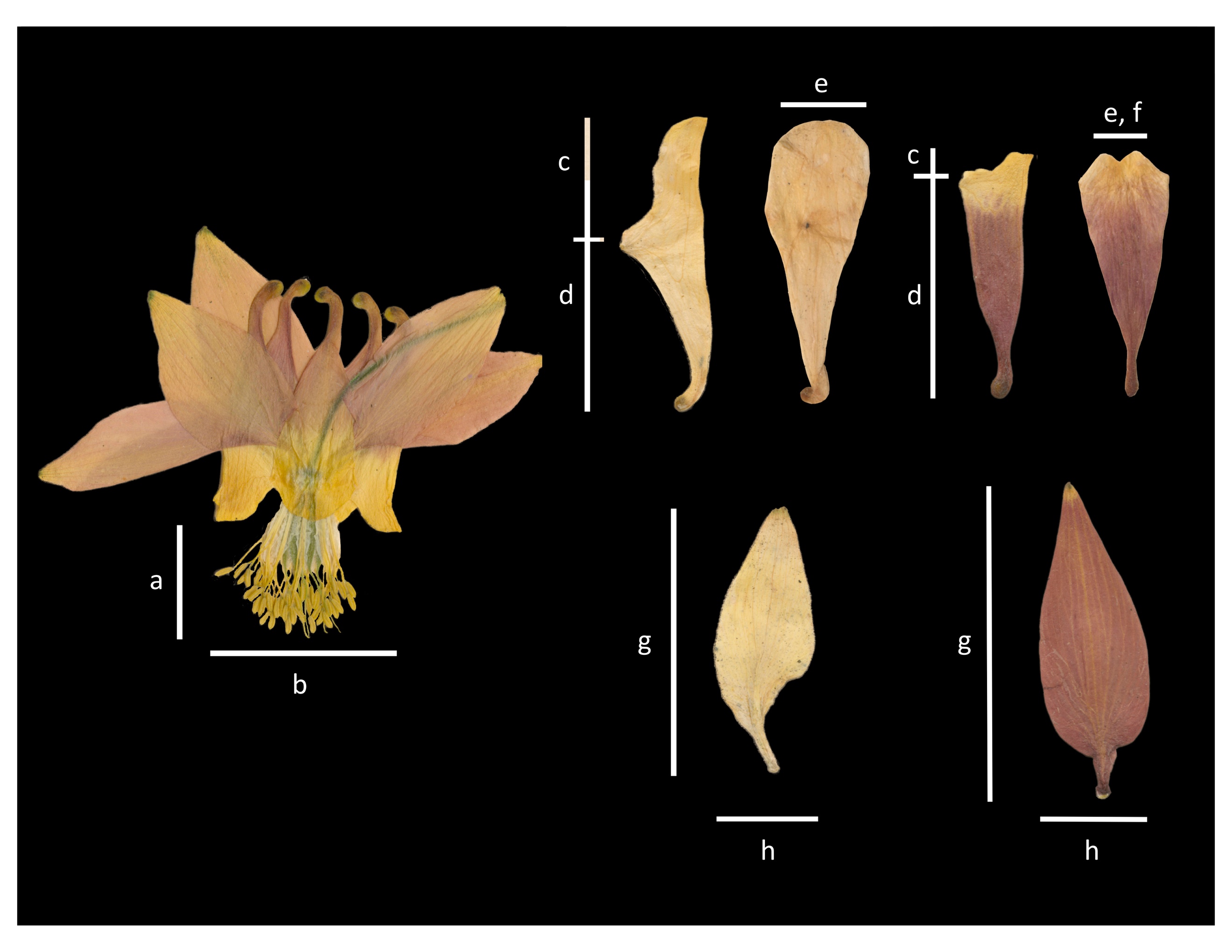


**APPENDIX S1** Diagram of floral characters measured from pressed Aquilegia specimens. (a) anther exsertion; (b) corolla width; (c) lamina length; (d) spur length; (e) lamina width; (f) cleft lamina; (g) sepal length; and (h) sepal width. Left: complete flower from an A. flavescens × formosa individual; middle: representative petals and sepal of A. flavescens; right: representative petals and sepal of A. formosa.

**APPENDIX S2** Relationship between collection date (a proxy for flowering time) and altitude. Yellow, Aquilegia flavescens; red, A. formosa. Shown is the regression line with 95% confidence bands for both species combined. The regression line intercepts for each species are not significantly different (F1, 169 = 0.31, P = 0.58). The combined slope estimate is 1.77 days/ 100 m (95% CI = 1.31, 2.22). Data are from UBC and Idaho Stillinger Herbarium collections.

**APPENDIX S3** Hybrid A. flavescens x formosa plants from the Marble Range, BC, on average show intermediate trait values with respect to allopatric populations of A. formosa and A. flavescens, with the exception of sepal length, a trait which is highly plastic within species. Yellow, A. flavescens from Mt. Kobau, sample size = 20; pink, A. flavescens x formosa from the Marble Range, BC, sample size = 32; red: A. formosa from near Clearwater, BC, sample size = 58.

| **Trait 1** | **Trait 2** | **Correlation (*r*)** | **Significance (P)** |
| --- | --- | --- | --- |
| corolla width | lamina length | 0.48 | 0.0052 |
| corolla width | lamina width | 0.71 | < 0.0001 |
| corolla width | sepal length | 0.46 | 0.0074 |
| corolla width | sepal width | 0.53 | 0.0020 |
| spur length | sepal length | 0.37 | 0.0360 |
| lamina width | sepal length | 0.40 | 0.0242 |
| lamina width | sepal width | 0.49 | 0.0041 |
| sepal length | sepal width | 0.75 | < 0.0001 |

**APPENDIX S4** Statistically significant pairwise floral trait correlations in Marble Range hybrid plants. Correlations between traits may reflect pleiotropy, linkage, or selection.


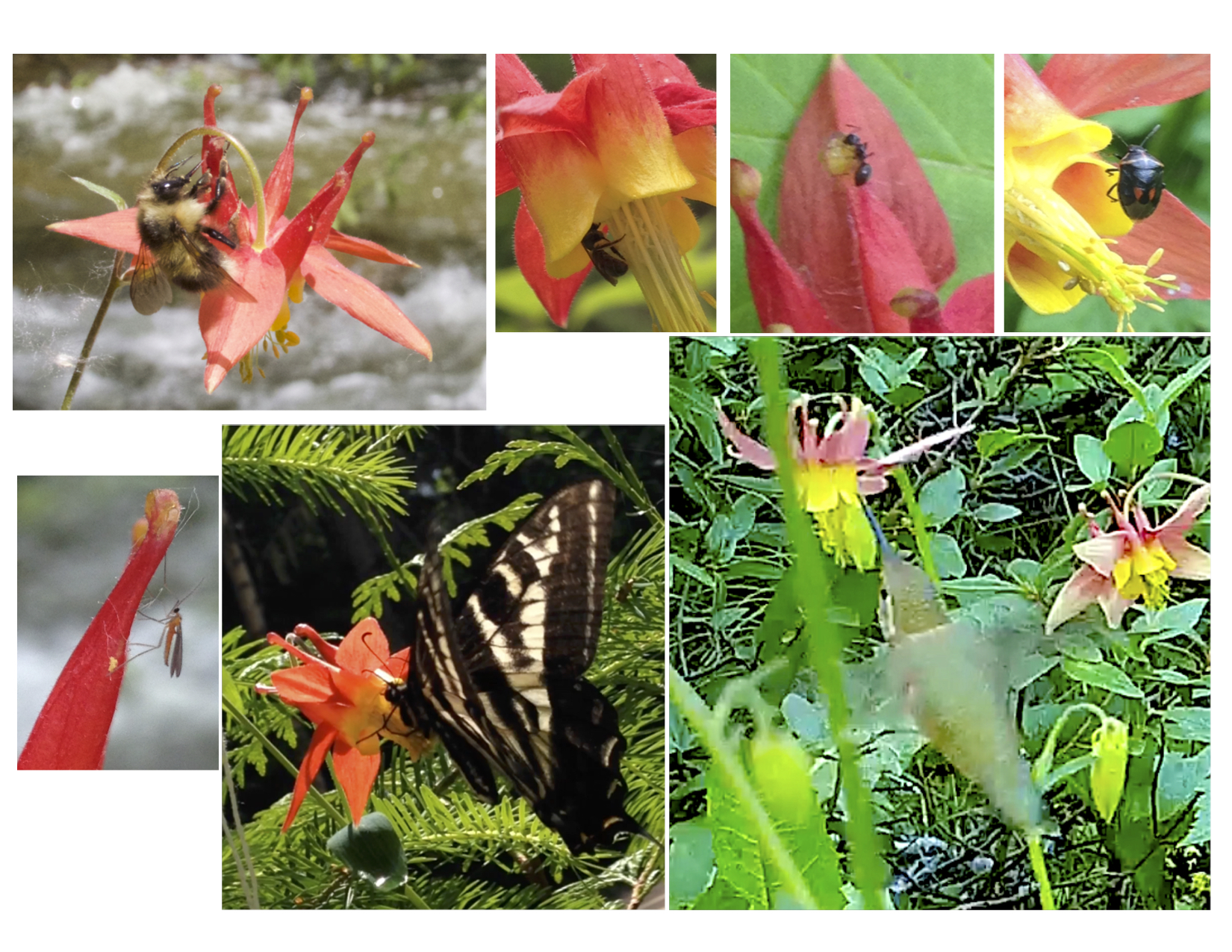


**APPENDIX S5** Visitors to A. formosa observed during the course of this study. Not all are pictured. See Appendix S8 for a video recording of hummingbird visitation.

**APPENDIX S6** Principal coordinate analysis implemented on a genetic distance matrix (Bruvo’s distance) of microsatellite genotypes of A. formosa, A. flavescens, and hybrids from the Marble Range. Yellow triangles, A. flavescens from Mt. Kobau; pink crosses, A. flavescens × A. formosa hybrids from the Marble Range, BC; red circles, A. formosa from Robert’s Lake, BC; red diamonds, A. formosa from near Clearwater, BC.

**APPENDIX S7** NJ (left) and UPGMA (right) trees constructed from a distance matrix (Bruvo’s distance) of microsatellite genotypes of A. formosa, A. flavescens, and hybrids from the Marble Range.

**APPENDIX S9** Comparison of nectar spur lengths of Aquilegia formosa and A. flavescens with the bill lengths of hummingbird species whose breeding ranges overlap with the ranges of the Aquilegia species. As the anthers of Aquilegia contact the gorget or forehead of hummingbirds, and the nectar may be reached by extension of a bird’s tongue, these comparisons may not precisely reflect Aquilegia pollen placement on hummingbirds.
